# Hierarchy of hair loss stigma: media portrayals of cancer, alopecia areata, and cancer in Israeli newspapers

**DOI:** 10.1186/s13584-019-0338-0

**Published:** 2019-09-03

**Authors:** Daphna Yeshua-Katz, Shifra Shvarts, Dorit Segal-Engelchin

**Affiliations:** 1 Department of Communication Studies, Ben-Gurion University of the Negev, POB 653, Beer-Sheva, Israel; 2 Moshe Prywes Center for Medical Education, Faculty of Health Sciences, Ben-Gurion University of the Negev, Beer-Sheva, Israel; 3 Spitzer Department of Social Work, Ben-Gurion University of the Negev, Beer-Sheva, Israel

**Keywords:** Treatment-induced alopecia, Alopecia areata, Agenda-setting, Framing, Stigma, Media representation, health policy

## Abstract

**Background:**

Over 300,000 people in Israel cope with temporary or permanent hair loss (alopecia) that results from diseases and medical treatments. For women, hair loss can be a highly traumatic event that may lead to adverse psychosocial consequences and health outcomes. Nevertheless, this phenomenon has been mostly ignored by health professionals as it is primarily considered an aesthetic—rather than as a health-related issue. Only recently the Healthcare Basket Committee approved financial assistance for the purchase of wigs by patients coping with hair loss. Given the important role that the media plays in shaping health policies related to diagnoses, treatment and support services, the current study sought to enrich our understanding of how the media portrays disease-related hair-loss.

**Methods:**

Using framing and agenda-setting theories, this study examined the media portrayals of hair loss associated with three diseases—cancer, alopecia areata, and ringworm, depicted in Israeli newspapers in 1994–2016. The sample consisted of 470 articles about the three diseases: 306 on cancer, 36 on AA, and 128 on ringworm.

**Results:**

Textual and visual analysis revealed the ways media marginalize this physical flaw. Cancer was framed in medical terms, and patients were portrayed as older Israeli-born people whose hair loss was absent from their experience. Ringworm was framed as a fear-inducing disease; patients were portrayed as faceless, unidentified immigrants that coped with visible hair loss. Articles on AA provided the greatest focus on the patient’s experience of hair loss, but patients were portrayed as young foreign people.

**Conclusions:**

Our results revealed a hierarchy of stigmas against hair loss, in which the media coverage marginalized this experience. The omission of hair loss by the media may explain, at least in part, why health professionals often ignore the psychosocial needs of these patients. Health insurance funding of wigs is a helpful but nevertheless insufficient solution to coping with feminine hair loss. Our findings may encourage media leaders to conduct planned media interventions to increase awareness of clinicians and health policymakers about the unique challenges faced by women coping with hair loss and promote health policy-making aimed at the well-being of these women.

## Background

Hair is a major aspect of body image, and it plays a role in social interactions. For women, in particular, hair is an important indicator of personality, attractiveness, and femininity [1]. Hair is a public marker of ancestry, age, general health, and sexuality [2]. Cross-cultural studies [1, 3–5] that examined women diagnosed with breast cancer found that hair loss was a traumatic event, sometimes even more traumatic than the loss of a breast [6]. Nevertheless, the female hair loss phenomenon has gained little medical awareness, because it is not a life-threatening condition and has an indirect health impact. As a result, it is classified mainly as an aesthetic, and less as a medical issue.

In January 2019, after 4 years of repeating rejections, the Israeli Healthcare Basket Committee has finally approved financial support for hair loss. The committee approved the purchase of wigs for those who cope with hair loss due to Alopecia. This type of service is a helpful but an insufficient solution to coping with the psychosocial challenges of women suffering from hair loss. One of the factors that may impact the Israeli health policies preferences is the media coverage of these health conditions. The media attention to and construction of health-related issues contributes in significant ways to health policies related to diagnoses, treatment, prevention, health promotion, research directions, and support services [7–17].

To understand the lack of health policy support to female hair loss in Israel, this study examines the ways the Israeli media defines, frames, and represents disease-related hair loss of AA, ringworm and cancer as reported in Israeli newspapers. The findings make it possible to gain insights about the nature of hair loss stigma and its nuanced relationship with disease context. More broadly, this study contributes to our understanding of how the public image of hair loss—that may impact health policy preferences—is constructed by the media.

## Theories of media analysis

Mass media articles shape and reflect public and cultural images. These images can lead to the stigmatization of individuals with diseases. There are many useful frameworks to describe portrayal and description of diseases by the media, such as Leventhal’s common sense model (CSM) [18], that explicates patients’ representations of illnesses and treatments from other environmental cues such as mass media or the framework of terminology used in representing disability [19–21] in the media coverage. In this study, we chose to use the agenda-setting theory [8], which posits that the frequency and prominence of a disease in the media impacts attitudes toward the disease and influences the social agenda regarding health issues related to the disease. By producing, repeating, and reinforcing words and images that convey some ideas, but not others, it is thought that the media makes some events and issues more salient than others [9]. The second relevant theoretical framework is framing [10, 22]. Framing posits that the media selects some aspects of a perceived reality and makes them more salient, with the intention of promoting a particular definition, interpretation, moral evaluation, and/or treatment recommendation for the item described [10]. The framing and salience of an event or finding in the media can systematically affect how recipients come to understand the news [11]. Framing also allows recipients to consider the importance of an item, because the media actively sets the frame of reference, which the audience then uses to interpret and discuss public events [23]. When journalists frame a message, they connect a topic to notions that are part of the “common ground” within a given culture, such as its values, archetypes, and shared narratives [24, 25]. Thus, it is possible that communicators that transmit the dominant messages regarding cancer, ringworm, and AA are not fully conscious of the long-term effects that they create by constantly repeating and reinforcing the same mental images [12].

Through the processes of agenda-setting and framing, the media may reflect an established hierarchy of chronic diseases and prestige rankings of disabilities [13] by marginalizing or prioritizing certain health conditions and groups of patients. In turn, these processes will perpetuate social and political power differentials with regard to health-related issues.

In several cases, the media has either prioritized or marginalized diseases and patients. For instance, breast cancer receives greater media coverage compared to other cancers [14]. Champion and colleagues [9] found that a “typical” patient with breast cancer was portrayed in the media as a young, Caucasian, “optimistic fighter”; in contrast, a typical patient with a heart or stroke condition is presented as though he/she is directly responsible for his/her own health decisions. In addition, patients with heart and stroke conditions that do not comply with lifestyle changes are sometimes framed as at fault for their condition, and they are “called out” by the media for their poor health choices [9]. Another case of media stigmatization of patients is media portrayals of severe mental illness patients suggest that the news media representation of persons with severe mental illness as violent may contribute to negative public attitudes towards this vulnerable population [15].

## Media and health policy

The impact of media portrayals of health issues on health policy decisions has been well documented in public policy research. Media serves mainly as a *contributor* in the policy process, with media accounts supplying consistent policy beliefs with matching narrative framing strategies to construct a policy story [26]. Media scholars have argued that it is important to understand the ways in which journalistic framing of health issues occurs, because framing influences public understanding and, consequently, policy formation [27–29]. Media are more than a mirror on which public policy players illuminate their messages; rather, the media are the uncredited directors of policy dramas. It is the media who hold the power to interpret the performance of political actors and remold the play’s structure [30, 31].

Media advocacy literature identifies two major areas in which the media has influenced health policy decisions: setting the agenda for health professionals and funding decisions. For example, media coverage of a Chicago hospital’s campaign, based on follow-up medical examinations of patients who underwent radiation treatment during childhood, had a snowball effect that prompted more medical institutions to follow suit, resulting in the National Cancer Institute (NCI) launching a nationwide campaign to warn the public and medical community about the late health effects of ionizing radiation [32]. An additional example is reflected in the negative media coverage of the HPV vaccine in Japan, contributing to the Japanese government’s reluctance to re-introduce HPV vaccination as an active recommendation [33].

The important role played by the media in influencing disease-specific funding decisions was documented in several cases. The amount and framing of news coverage of autism from 1996 to 2006 in the US helped push autism onto the national agenda prior to the passing of the Combating Autism Act, authorizing nearly $1 billion over 5 years to fund autism research and related activities [34]. Pressure from the Israeli media in the form of sensational coverage of ringworm patients permeated the Israeli parliament and led to the enactment of the Ringworm Compensation Law in July 1994, compensating ringworm patients that underwent radiation treatments and developed diseases [35]. Breast cancer funding activists used culturally resonant media frames to persuade audiences and to redefine breast cancer from a private problem of individual women to a major public health problem worthy of increased federal funding [36].

## The three diseases

Illness is a socially constructed phenomenon based on the value of life and health. It represents a deviation from what we hope for and expect [37]. However, some illnesses are imbued with additional cultural meaning that results in a “spoiled identity”, or a stigmatization of the individual [38]. Previous studies have shown that individuals with hair loss experience a stigma that they must deal with during social interactions. In turn, these stigmatizing experiences may lead to additional physical and mental health problems. Their stigma—an attribute which is socially discrediting [39]—is instantly visible when the hair loss is apparent, but hidden when they conceal their hair loss, for example, by wearing a wig [5]. We have selected the major diseases that are associated with hair loss, either as a direct result of the disease itself (e.g. AA) or by the medical treatments related to the disease. Nevertheless, although the three groups included in the present study share the same physical blemish, their illnesses have distinct social constructions, and they are associated with distinct cultural images.

### Alopecia Areata

AA is a benign inflammatory autoimmune disease prevalent in 2% of the population, which is characterized by the loss of hair without scars [40]. The etiology and subsequent development of alopecia are not fully understood, but it is an autoimmune disorder that arises from a combination of genetic and environmental influences [41]. Although it is not life-threatening and creates no significant pain, ache, or itching, it has been associated with notable emotional stress, low self-esteem, depression, and anxiety [42]. Women with AA reported a worse quality of life compared to men with AA [43]. Patients with AA have reported higher levels of self-stigmatization than patients with mental illness [44].

In Israel, there are currently 323,751 AA patients (110,929 men, 212,822 women) according to the Department of Quality Measures and Research of Clalit Health Services, which is the largest health services provider in Israel (Arnon Cohen, Chief Physician Office, General Management, personal communication, January 17th 2019). Despite this large number of patients with AA in Israel, the disease is not well known among the public. Therefore, a person with AA is typically perceived as a person with cancer that underwent chemotherapy. One of the health services available covers special populations and provides assistance in financing various rehabilitation devices. However, despite assistance for common aesthetic rehabilitation devices, like dentures and stump socks, no assistance is available for an aesthetic rehabilitation device for baldness. Only in 2019, after 4 years of repeating requests, wigs for patients who cope with AA were approved by the Health Services Basket Committee, in addition to new drugs and technologies,

### Ringworm

Ringworm of the scalp (tinea capitis) is a highly contagious fungal skin disease that primarily affects children. It tends to start out as a bump or small sore, it might turn flaky and scaly, and it leads to patches of hair loss. For centuries, ringworm was treated by manually plucking out the infected hairs of children. In 1910, following the discovery of X-rays by Roentgen, ringworm began to be treated with low dose radiation, which eliminated the infected hair. Currently, ringworm can be treated with a prescription medication.

From ancient history, ringworm was a stigmatic disease, due to the ugly wounds and scars it caused on the head; it was often called ‘scald-head’, and children with ringworm were often excluded from schools and society. In Israel, there are nearly 6000 women with partial or complete hair loss due to irradiation for ringworm in childhood [45]. Radiation therapy has been the standard care for children with ringworm, since 1925 in Israel. In the 1950s, during mass immigration to the country, the Ministry of Health sent individuals with ringworm (mostly children, but also immigrants, veterans, Jews, and Arabs) to public X-ray clinics, where they received radiation therapy [46]. The treatment resulted in baldness, which was considered shameful. All irradiated children experienced temporary baldness after treatment (for a six-week period); 5% remained completely bald for the rest of their lives; and about 14% remained partially bald for the rest of their lives [47]. In 1974, Baruch Modan, then a prominent Israeli epidemiologist, led a research team that studied the high prevalence of head and neck tumors among people which, as immigrant children in Israel, were irradiated for ringworm in the 1950s [48]. This seminal work had two consequences: first, it placed the issue of ringworm treatment on the public agenda in Israel; and second, it was followed by political activism that led to the 1994 Ringworm Victim Compensation Law, which stated that people that had been treated for ringworm in the 1950s were entitled to monetary restitution from the state.

In addition to the social stigma, radiation therapy for ringworm appeared to have dire psychosocial consequences and severe health outcomes,. A recent follow-up study of women in Israel treated with radiation for ringworm in childhood found a high prevalence of social abuse social anxiety, depression and migraines [47]. That study also revealed high rates of psychiatric medication use and hospitalizations in mental health institutions.

### Cancer

In Israel, about 4500 women are diagnosed with breast cancer each year [49], with many experiencing chemotherapy-induced alopecia. In contrast to the mechanisms of the stigma associated with ringworm and AA, the stigma associated with cancer is thought to be driven primarily by fear of the illness. Cancer has been described as the most feared of modern diseases [24, 50, 51]. Fear has led to the stigmatization of individuals with cancer, and they are isolated from social life [38, 50]. This stigmatization has given rise to concerns about the reluctance to disclose patient medical histories for occupational and social purposes. Occupational stigmatization frequently arises from the myths that cancer is a death sentence; that cancer survivors are unproductive, and thus, a drain on the economy; and that cancer is contagious [52]. The social stigma of females with cancer is also driven by treatment-induced hair loss. Women across cultures often report that hair loss is one of the more troublesome results; it makes them feel like they look unattractive, sick, or dying. Furthermore, they often feel stigmatized by others [2].

Although cancer is associated with severe physical limitations and negative experiences, it is not associated with social groups considered to be morally guilty. On the contrary, media portrayals of individuals with cancer often describe them with “imagined superpowers,” [53] which illustrates the common goal of self-willed victory over cancer and any limitations of the body. In fact, women with breast cancer are openly honored as “survivors” [54] and heroic [53].

Although individuals with the three diseases that cause baldness must cope with a similar physical flaw, they received different levels of compensation for their baldness. Patients with ringworm are compensated by the Israeli 1994 Ringworm Victim Compensation Law, when they have a disease listed in the addendum to the law. Patients with cancer receive assistance in buying wigs during their treatment period, through many dedicated associations. Although more than 50,000 people are diagnosed with AA in Israel, only recently, 23 years after the National Health Insurance Law came into effect, the Healthcare Basket Committee has decided to include financial assistance for purchasing wigs for patients with AA in the healthcare basket. Nevertheless, the Israeli healthcare system continues to ignore the psychosocial aspects of hair loss, as it is mainly classified as an aesthetic issue.

Taking a broad view of the epidemiological, historical, and social meaning of each of these three causes of hair loss, it is important to understand the way the media—a major source of health information for health professionals and the public at large—defines, frames, and represents the three diseases. Therefore, the present study used the agenda-setting and framing theories to examine and compare the media coverage of ringworm, AA, and cancer in Israeli newspapers. In the tradition of inductive research, we gleaned understanding and meaning by carefully reading and analyzing the text and images of diseases, as described. Thus, the specific aims of the current study were to: (1) identify the dominant frames related to ringworm, AA, and cancer in Israeli newspapers; (2) examine the dominant media portrayals of patients with ringworm, AA, and cancer in Israeli newspapers; and (3) examine the salience of hair loss experiences reported in Israeli newspapers in describing patients with ringworm, AA, and cancer. Understanding media portrayals of diseases that cause hair loss is crucial, because these portrayals serve as a window into how society understands induced hair loss, its causes, and its social implications.

## Method

### Data collection

This study was based on a disproportionate, stratified sample of every article on ringworm and AA and randomly selected (every 40th) articles on cancer from the 20 leading newspapers, published in 1994–2016. To achieve an appropriate sample size, we used six search engines: *Yedioth Ahronont’s* digital archive; the *Ha’aretz* digital and print archive; the *Bet Ariela* dailies bibliographic database; and three online news editions (*Ynet*, *NRG*, and *Mako)*. These six search engines were selected, because they provided access to the most popular circulating print and online editions of national and local newspapers. We selected newspapers with the highest readerships that were distributed nationally, locally, and online, These included four national print editions (*Yedioth Aharonot*, *Maariv*, *Ha’aretz*, and *Hadashot*), two print editions of religious dailies (*Hatzofe* and *Yated Ne’eman*), 11 print editions of local newspapers, and three online news editions (*Ynet*, *NRG*, and *Mako*). This research did not involve human subjects and therefore did not require IRB approval.

We sampled only a fraction of articles on cancer to limit the sample size to a manageable number for in-depth quantitative analyses and to achieve a sample size similar to the total media representation of ringworm and AA. We chose the time frame of 1994–2016 to avoid the bias of reporting on only one year, where one story dominated, such as that of the 1994 Ringworm Victim Compensation Law, and to ensure a sufficient number of articles on the topic of AA, which was a relatively new disease. We employed the search words: “cancer,” “ringworm,” and “alopecia areata,” and we set the time frame to be inclusive (i.e., from January 1994 to December 2016). Articles were included when they only mentioned one of the three diseases or they discussed the diseases along with another topic. The final sample consisted of 407 articles about the three diseases: 306 on cancer, 128 on ringworm, and 36 on AA.

### Procedure

Each item was catalogued with coding that was developed during past research. We gathered information for descriptive purposes (e.g., type of disease; title; author; date; and local, national, or online source). We also searched for textual content that pertained specifically to the way patients were represented (or neglected) and their hair loss experience. These categories included (a) patients (number of patients interviewed); (b) hair loss description (numbers of words in each item); and (c) hair loss coping efforts (number of words devoted to descriptions). We assumed that, when more space was devoted to a particular disease aspect, it indicated a greater emphasis on that aspect, on the part of media [9]. Patient representations were coded in terms of (d) patient gender, based on previous research that indicated that gender influenced media portrayals of patients [9, 53], and (e) birthplace, because a stigma was associated with immigrant populations [55]. It remains unknown whether birthplace is related to cancer and AA.

We also analyzed visual portrayals of patients, because images capture attention more readily than text [9]; moreover, visual images influence recall and the comprehension of information [56]. Therefore, we counted (f) the number of images per article; (g) the number of people displayed per article; and (h) the number of patients displayed per article. We also coded images in each item according to the depiction of (i) visible hair loss to examine the prevalence of images of the visual impact of disease/treatment on the body [56, 57]; and (j) obscured faces in patient images, because concealing a patients’ identity reflects dehumanization [58] and the social stigma [39].

### Framing

A coding scheme for the framing analysis was developed inductively, as the researchers read and discussed the articles. The first stage involved reading, rereading, discussing, and then organizing the raw data into three conceptual framing categories, based on Clarke and Everest’s [50] and Semetko and Valkenburg’s [59] frames. We included four frames: (a) political portrayals of disease as originating in causes that lie outside the individual; for example, diseases caused by environmental contaminants or medical treatments. We also included references to advocacy and compensation efforts from the state/institutions (Clarke & Everest, 2006); (b) medical depictions of disease as a physiologically-based pathology that could be explained and discussed within medicine (Clarke & Everest, 2006); (c) human interest, which brings a human face or an emotional angle to the presentation of an event, issue, or problem [59]; and (d) fear, which implements the language of fear and panic derived from an ongoing epidemic or public health crisis; in addition, exaggerated and unverified statistics might be used [60, 61]. The preliminary framing categories became clear, after approximately half of the articles were read. Subsequent reading codified articles into the emergent categories, which were added to or refined, as the reading and coding progressed. In the case of a cross-over of frames, for example an article with a political portrayal of the disease that also mentions fear as a main factor, the four coders discussed the coding of the article until they reached an agreement on the article’s main frame.

### Coding and reliability

Four researchers were extensively trained during a three-month period to become familiar with all of the definitions in the codebook and to practice coding articles. Following the initial training, these coders began the coding process. Reliability was checked every month on a subset of articles that all four coders had rated. Overall, 200 articles (49% of the sample) were coded by all four coders, and they were used for the reliability analyses. Reliability for each of the individual variables was calculated with Krippendorff’s Alpha (α). Tables 1, 2, 3, 4 and 5 show the final coefficients for each variable.

**Table 1:** Prevalence of media coverage for different disease types

Coverage	Ringworm	AA	Cancer	α	χ^2^ Value	LB 95% CI	UB 95% CI
References to disease/patients	11.9%^a^	55.3%^b^	3.2%^c^	0.94	102.07***	.0001	.0001
Distribution							
Newspaper, national	60.6%^a^	68.4%^a^	87.5%^b^		67.49***	.0001	.0001
Newspaper, local	10.1%^a^	0.0%^b^	0.4%^b^				
Online news	20.2%^a^	31.6%^b^	11.4%^c^				
Religious newspapers	9.2%^a^	0.0%^b^	0.7%^b^				
Story type							
News	75.2%^a^	57.9%^b^	80.4%^a^	0.79	19.40***	.001	.002
Opinion	11.9%^a^	7.9%^b^	3.6%^b^				
Magazine	12.8%^a^	34.2%^b^	16.1%^a^				

*AA* alopecia areata, α Krippendorff’s Alpha, *95% CI* 95% confidence interval, *LB* lower bound value, *UB* upper bound value

Different superscript letters indicate significantly different prevalence. ***p < .001

**Table 2:** Prevalence of framing for different disease types (α = 0.76)

Frame	Ringworm	AA	Cancer	χ^2^ Value	LB 95% CI	UB 95% CI
Political	75.8%^a^	35.7%^a,b^	10.5%^a^	194.41***	.0001	.0001
Medical	11.6%^b^	28.6%^b^	71.7%^b^			
Human-interest	4.2%^b^	35.7%^c^	17.8%^c^			
Fear	8.4%^a^	0.0% ^a,b^	0.0%^a^			

*AA* alopecia areata, *95% CI* 95% confidence interval, *LB* lower bound value, *UB* upper bound value

***p < .001. Different superscript letters indicate significantly different prevalence

**Table 3:** Prevalence of hair loss mentioned for different disease types

Salient factors	Ringworm	AA	Cancer	α	χ^2^Value	LB 95% CI	UB 95% CI
Hair loss mentioned	34.9%^a^	44.7%^a^	10.1%^b^	0.90	163.93	.0001	.0001
Coping efforts mentioned	18.3%^a^	31.6%^b^	6.1%^c^	0.76	47.43	.0001	.0001
						F_(2, 21.47)_	ɳ^2^
Weighted word count, mean	8%^a^	55%^b^	12%^a^			22.80***	.49
SD	0.09	0.36	0.14				

*AA* alopecia areata, α Krippendorff’s Alpha, *95% CI* 95% confidence interval, *LB* lower bound value, *UB* upper bound value

Different superscript letters indicate significantly different prevalence. ***p < .001

**Table 4:** Patient representations in text according to disease type

Characteristic	Ringworm	AA	Cancer	α	χ^2^ Value	LB 95% CI	UB 95% CI
Gender							
Female	15.6%^a^	26.3%^b^	25.7%^b^	0.90	38.62	.0001	.0001
Male	20.2%^a^	10.5%^b^	5.7%^b^				
Female and Male	12.8%^a^	0.0%^b^	3.2%^b^				
No indication	51.4%	63.2%	65.4%				
Birthplace							
Israel	3.7%^a^	2.6%^a^	31.1%^b^	0.83	4.12	.37	.39
Other	22.9%^a^	28.9%^a^	1.4%c				
No mentioned	73.4%	68.4%	67.5%				
Number of patients interviewed					0.76	F_(2, 141.51)_	ɳ^2^
mean	1.10	1.18^a^	0.68^b^			3.20***	.02
SD	2.16	0.90	1.37				

*AA* alopecia areata, α Krippendorff’s Alpha, *95% CI* 95% confidence interval, *LB* lower bound value, *UB* upper bound value

Different superscript letters indicate significantly different prevalence. ***p < .001

**Table 5:** Patient representations in images according to disease type

Characteristic	Ringworm	AA	Cancer	α	χ ^2^ Value	LB 95% CI	UB 95% CI
Hair loss	13.8%^b^	23.7%^a^	4.6%^c^	0.88	29.85***	.0001	.0001
Obscured face	11.0%^a^	5.3%	2.1%^b^	0.88	16.96***	.002	.004
						F_(2, 141.51)_	ɳ^2^
Number of images, mean	0.71^a^	1.65^b^	1.56^b^	0.85		53.21	.04
SD	0.99	1.58	2.11				
Number of people displayed, mean	3.24	1.67	2.38	0.76		1.19	.00
SD	5.18	1.07	5.69				
Number of patients displayed, mean	1.05	1.36	1.49	0.77		0.52	.00
SD	1.43	0.92	6.00				

*AA* alopecia areata, α Krippendorff’s Alpha, *95% CI* 95% confidence interval, *LB* lower bound value, *UB* upper bound value

Different superscript letters indicate significantly different prevalence. ***p < .001

### Data analysis

The effect of the disease type (ringworm, AA, or cancer) on media-related categorical outcome measures was examined with chi-square tests for independence of measures and with the Monte Carlo resampling technique to assess significance (10,000 resamples). The effect of the disease type on media-related quantitative outcome measures was examined with one-way analyses of variance (ANOVA). Since the analyses involved different sizes of samples we used two recommended statistical tests to adjust the analyses for inequality of variances: Brown-Forsythe correction [62] and with Tamhane posthoc [63] tests.

## Results

### Media coverage

In this section, we examined whether the disease type affected the prevalence of articles written about the disease and/or the patients. We also examined the distribution of media that covered the disease and/or the patients (national, local, online news, or religious newspapers). The results are summarized in Table 1.

Table 1 shows that significantly more articles on cancer (approximately 90%) were printed in national newspapers compared to ringworm and AA (approximately two-thirds). The remaining articles on cancer and AA were mostly published online. In contrast, articles on ringworm were evenly distributed between local newspapers, religious newspapers, and online news sites.

### Framing diseases

Next, we examined the different ways that diseases were framed (political, medical, human-interest, and fear). The results are summarized in Table 2.

Table 2 shows that most articles on ringworm were framed as political issues, and those on cancer were framed as medical issues. Articles on AA had no unique framing, but were spread across all framing categories, except fear. Ringworm were the only disease that was framed as a fear issue.

### Salience of hair loss

In this section, we examined whether the disease type affected the prevalence of articles that mentioned hair loss or hair loss coping efforts in the text. In addition, we examined differences in the weighted counts of words related to hair loss. The results are presented in Table 3.

Table 3 shows that significantly fewer articles mentioned hair loss or coping efforts related to cancer, compared to articles about both ringworm and AA, and significantly more articles mentioned coping efforts related to AA, compared to articles about ringworm. In addition, more than half of the words in the articles about AA were about hair loss, compared to approximately 10% of the words in articles about ringworm or cancer.

### Patient representations in text

In this section, we examined whether the disease type affected the number of patients interviewed, their gender, and their place of birth. We also examined whether the patient covered was a celebrity. The results are summarized in Table 4.

Table 4 shows that fewer patients with cancer were interviewed, compared to patients with AA (the number of patients with ringworm interviewed was not significantly different from either group). More articles about cancer neglected to mention patient ages (above or below 18 y) and gender, compared to articles about ringworm (the difference was marginal for AA). High percentage of the articles (Ringworm - 51.4%, AA - 63.2% and cancer - 65.4%) had no indication of the gender. Thus, the descriptions of the patients based on the analysis relates to both male and female patients.

Israeli-born patients were represented significantly more frequently in articles about cancer compared to articles about AA or ringworm. Conversely, patients born in other countries were mentioned significantly more frequently in articles about AA and ringworm, compared to articles about cancer. Finally, celebrities were interviewed in significantly more articles about cancer, compared to articles about ringworm (AA was not significantly different from either of the other groups).

### Patient representations in images

In this section, we examined whether the disease type affected the total number of images in the articles, the total number of people displayed, the total number of patients displayed, the prevalence of patients with hair loss, and the prevalence of patients with obscured faces. The results are summarized in Table 5.

Table 5 shows that images of patients with visible hair loss were more frequently included in articles on AA than in articles on ringworm or cancer, and they were more frequently included in articles on ringworm than in articles on cancer. Conversely, images of patients with obscured faces were more frequently included in articles on ringworm than in articles on cancer (the frequency in articles on AA did not differ from either of these groups). Finally, articles on ringworm included fewer images than articles on AA and cancer. Other characteristics were not significantly different among the different diseases studied.

## Discussion

The current study was the first to compare media portrayals of a physical flaw (hair loss) caused by three distinct diseases—, AA, ringworm and cancer—in select circulating Israeli newspapers. Our findings revealed a hierarchy of stigmas against hair loss [13], in which the media coverage marginalized hair loss experience. Our content analysis showed that hair loss and each disease were given meaning and carried attributions in the media. Hair loss was not perceived as a purely cosmetic issue; rather, it was interpreted in terms of the available sociocultural and political meanings. In turn, these representations might influence public knowledge and attitudes, as well as the position of the health care system and doctors and nurses, towards women suffering from baldness.

Consistent with previous research [64], we found that, for each disease type, the media presented both biased and “typical” images of patients and their efforts to cope with hair loss. Importantly, our findings revealed the ways media marginalize each of the studied group. In turn, an Israeli women who is facing hair loss may feel stigmatized due to lack of media representation and discourse around of this physical flaw.

The person with ringworm, regardless of gender, was portrayed as an immigrant that must cope with visible hair loss. These patients were less likely to be interviewed and, when interviewed, appeared faceless and unidentified. This finding suggested that people with ringworm might face social exclusion, both as immigrants and as victims of a contagious disease. Consequently, a person with ringworm might feel stigmatized, due to the connection between ringworm and an immigrant population, and isolated, due to the fear of contagion. The fear of getting too close to a person with ringworm can affect relationships with intimate, significant, and more distant others (i.e., medical personnel). In addition to stigmatizing the patients, the media did not frame the disease in medical terms, but rather, in terms of political struggles and fear of contagion.

People with AA were portrayed in the media as foreign patients that are vocal about their hair loss experience. They were identified in both text and images, and their hair loss was visibly depicted. Unlike ringworm, AA was not framed in any particular way, but the patient’s experience was likely to be central to the story. Although data from the largest Israeli HMO indicates that over 300,000 people were diagnosed with AA in Israel, the media portrayals gave the impression that the disease did not exist in Israel. These findings implied that a person with AA was likely to feel stigmatized, due to hair loss, and might be convinced that they were alone in coping with AA.

People with cancer were portrayed in the media as Israel- = born patients that identified themselves, both in text and in images. Nevertheless, although induced hair loss may be a traumatic event for patients with cancer, the illness experience and, in particular, the induced hair loss phenomenon, received less media attention than the hair loss among patients with AA. This portrayal of a person with cancer implied that, although hair loss was a traumatic event for some patients, it was portrayed as a minor issue in the overall illness experience.

The absence of people’s subjective experiences of disease- and treatment-induced hair loss in the media may explain, at least in part, the lack of attention given to the psychosocial needs of these people by health professionals. Given previous findings showing the important role that hair plays in shaping women’s well- being, health policymakers need to be particularly aware of the psychosocial challenges faced by women suffering from hair loss, and to develop policies that address their diverse needs.

## Conclusions

The mass media play an influential role in the process of agenda setting by calling attention to certain medical problems in a primary attention arena of the public domain and by framing what are seen as the causes and solutions to those problems [10, 13, 65]. The hierarchical media representation found for the three diseases considered in the present study might influence the public agenda and medical community by highlighting the experience of hair loss among people with cancer and AA, but silencing the hair loss experience of people with ringworm. In turn, this negligence might perpetuate the low social and political power connected to ringworm. These findings further supported the claim stated by Grue et al. [13] that agenda setting processes reflect a hierarchy of chronic diseases and prestige rankings of disabilities by marginalizing or prioritizing certain health conditions and groups of patients.

Our data did not provide clues to why this pattern of coverage occurred. It is possible that this pattern stemmed from the ringworm stigma rooted in the 1950s and 1960s, when ringworm was associated with immigration in the Israeli media. The coverage pattern might also be explained by the strong interest groups (e.g., researchers, health professionals, drug companies, etc.) involved in the prevention and treatment of cancer and by the lack of scientific understanding about the causes of AA.

This study had several limitations. First, the study compares media portrayals of hair loss associated with three diseases - cancer, AA, and ringworm. However, in the case of cancer and ringworm, the disease is of much greater severity compared to AA. The relative severity of the disease should be taken into account as a factor that may affect the salience of media attention to hair loss. In comparison - AA is, at its core, is about hair loss, such that media coverage is bound to focus more specifically on this aspect, and the health outcomes of AA are less severe than the other two diseases. Thus, there is a confounding factor at work here that may account for the observed findings. Second, our study did not include magazines oriented toward women readers. It is very possible that inclusion of those type of magazines would have yielded different findings. Third, our study was restricted to the Israeli media. Future studies would benefit from a cross-cultural examination of media portrayals of feminine hair loss. Future studies would also benefit from investigating how disease-related hair loss is portrayed in the social media. Additionally, future studies should examine media portrayals of other physical flaws that result from diseases and medical treatments. Lastly, qualitative studies are recommended to enrich our understanding of the agenda settings and framing processes that guide media professionals.

Despite the limitations, this study provides insight into the ways the media may impact health policies related to physical flaws caused by distinct diseases. The marginalization of people’s hair loss experience in the media, as found in this study, may encourage media leaders to conduct planned media interventions [66]— described as purposive activities that utilize diverse media channels to inform or motivate populations [67]—to increase awareness of clinicians, media professionals and health policymakers about the unique challenges faced by people, especially women, coping with hair loss. By using media interventions, then, media leaders may affect the different stages of health policy-making aimed at promoting the well-being of women coping with hair loss, including: agenda-setting, policy formulation, adoption, and implementation [68]. This may pave the way to the development of health policy geared to addressing the various effects of hair loss resulting from diseases and medical treatments among women.

## Data Availability

The datasets generated and analyzed during the current study are not publicly available due to paid access to newspapers archives but are available from the corresponding author on reasonable request.

## Abbreviation

AA: Alopecia areata
