# Correction to: Hierarchy of hair loss stigma: media portrayals of cancer, alopecia areata, and ringworm in Israeli Newspapers

**DOI:** 10.1186/s13584-019-0341-5

**Published:** 2019-10-14

**Authors:** Daphna Yeshua-Katz, Shifra Shvarts, Dorit Segal-Engelchin

**Affiliations:** 1 Department of Communication Studies, Ben-Gurion University of the Negev, POB 653, Beer-Sheva, Israel; 2 Moshe Prywes Center for Medical Education, Faculty of Health Sciences, Ben-Gurion University of the Negev, Beer-Sheva, Israel; 3 Spitzer Department of Social Work, Ben-Gurion University of the Negev, Beer-Sheva, Israel

**Correction to: Isr J Health Policy Res**

**https://doi.org/10.1186/s13584-019-0338-0**

The original publication of this article [1] contained an incorrect title. The correct and incorrect information are shown below:

- **Incorrect**: Hierarchy of hair loss stigma: media portrayals of cancer, alopecia areata, and cancer in Israeli newspapers
- **Correct:** Hierarchy of hair loss stigma: media portrayals of cancer, alopecia areata, and ringworm in Israeli Newspapers

The original publication has been updated.
